# Cloning, nucleotide sequencing, and bioinformatics analyses of growth hormone mRNA of Assaf sheep and Boer goats reared in Egypt

**DOI:** 10.1186/s43141-020-00046-6

**Published:** 2020-07-13

**Authors:** Waleid Mohamed El-Sayed Shakweer, Hashem Hamed Abd EL-Rahman

**Affiliations:** Animal Production Department, Agricultural and Biological Research, Division, National Research Centre, 33 El Bohouth St. (Former El-Tahrir St.), Dokki, Giza, P.O. 12622 Egypt

**Keywords:** Assaf sheep, Boer goats, Growth hormone sequence, Bioinformatics

## Abstract

**Background:**

Identification of molecular characterization of genes underlying livestock productive traits may allow applying advanced biotechnology techniques to improve animal productivity. Growth hormone (GH) controls body growth rate, milk production, reproduction as well as carbohydrate, lipid, and protein metabolism. Therefore, the present study aims to investigate the genetic variations of growth hormone cDNA sequences between Assaf sheep (As_GH) and Boer goat (Bo_GH) that mainly used for genetic improvement in Egypt using bioinformatics analysis. Growth hormone cDNA was isolated from the pituitary gland tissue of Assaf sheep Boer goat and subcloned into pTZ57R/T cloning vector for sequencing.

**Results:**

Molecular weight of As_GH cDNA was 665 bp and was 774 bp for Bo_GH cDNA. The complete coding sequences (CDS) of As_GH and Bo_GH were registered in the GenBank database under accession number (AC: MH128986 and AC: MG744290, respectively). High homology percentage was observed (99.5%) between AS_GH and Bo_GH protein sequences with one different amino acid in the As_GH protein sequence (Arg^194^). The protein sequence of As_GH has only one motif signature; Somatotropin_1 from 79 to 112 aa compared to Bo_GH protein sequences and GenBank database that had two motifs signature. The growth hormone cDNA sequence of Assaf sheep has a unique three single nucleotide polymorphisms (SNPs) (A^637^A^638^G^639^) that encodes for arginine (Arg^194^); this insertion mutation (AAG) was not found in the growth hormone cDNA sequences of Boer goat in the present study and GenBank database breeds. This mutation can be used to develop SNPs markers for Assaf sheep.

**Conclusions:**

GH sequence of Assaf and Boer goat is highly conserved and the homogeny in the codon region (99.5%). The Assaf sheep GH sequence has a unique three SNPs that may be used to develop SNPs markers for such breed. Further studies are needed to investigate the genetic variations of growth hormone gene in different sheep and goat breeds in Egypt and document the relationship between these variations and the productive performance of animals.

## Background

In Egypt, sheep and goats are important domestic animals reared either under desert conditions or Nile valley. Sheep and goats contribute about 6% and 4.5% of the total red meat produced, respectively (Statistics of Ministry of Agriculture, 2018). Identification of molecular characterization of genes underlying livestock productive traits may allow applying advanced biotechnology techniques to improve animal productivity. Growth hormone (GH) controls the body growth rate, milk production, reproduction as well as carbohydrate, lipid, and protein metabolism. The previous study demonstrated that the GH mRNA expression was significantly greater in all tissues examined in fertile Lezhi goat does than in infertile Tibetan goat does. This indicated that the GH may play an important role in the development of preovulatory follicles and the number of ovulatory follicles and finally, the ovulation rate in goat does [1]. The genetically selected sheep for low fatness have higher blood concentration of growth hormone than the other sheep [2].

The Assaf sheep and Boer goat breeds are widely used in Egypt for genetic improvement whether using artificial insemination or natural mating. Assaf sheep was produced by crossbreeding between Palestinian Awassi and German East Friesian sheep. It is a dual-purpose breed, raised for both milk and meat; however, it is used primarily for milk production [3]. It is worth mentioning that Assaf sheep are widespread in Spain, Portugal, and Middle East countries, but they are limited spread in Egypt. Assaf sheep adult body weight is about 110 kg for rams and about 80 kg for ewes. The growth rate of lambs is about 350 g/day from birth to 4 months. Age at first lambing is 13 months, and fertility ranged from 75 to 85%. Litter size is 1.4, and the average milk yield is 359 L in 220 days of lactation, with milk of 7.2% fat and 5.5% protein [4].

South Africa is the origin of Boer goat [5], and it is specialized for meat production. Boer goat is well adapted for tropical and semitropical conditions, high fertility rates, and resistance for the disease [6]. Boer goat is one of the most meat breeds spread in the world due to fast growth rate and excellent carcass qualities, where the average litter size is 1.9 kids/doe, the litter birth weight is 6.0 kg, and the average kid birth weight is 3.2 kg [7]. The present investigation aims to study the genetic variations of isolated growth hormone cDNA sequences from Assaf sheep and Boer goat breeds that used for the genetic improvement of small ruminant local breeds in Egypt.

## Methods

### Isolation of growth hormone and cDNA synthesis

The Boer goat was imported from South Africa by Egyptian Ministry of the Agriculture, while the Assaf sheep was obtained from Sinai and both have reared at National Research Centre farm from 2013, Noubaria, El-Beheira Governorate. For each breed, two pure males at third generation were slaughtered to obtain their pituitary gland tissues for GH mRNA isolation. The total RNA was isolated from the pituitary gland tissues of Assaf and Boer breeds using *Biozol kit* (Bioflux®) and reverse transcribed to the cDNA using *First Strand cDNA Synthesis Kit* (Fermentas, Canada).

### Polymerase chain reaction (PCR)

The efficiency of reverse transcription has been assessed using PCR. As_GH primer was published on NCBI (Ac: EU935861.1, 671 bp), As_GH-F 5′-GCTCACCAGCTATGATGGCTG-3′ and As_GH-R 5′-TGGCAACTAGAAGGCGCAGCT-3′**,** while Bo_GH primer was designed based on the National Center for Biotechnology Information (NCBI) database (Ac: X07035, 785 bp) using Primer-Blast tool available at https://www.ncbi.nlm.nih.gov/tools/pr imer-blast/. The Bo_GH primer sequence was Bo_GH-F 5′-CCGCGGAGGGTCCTGCTGACAGCTC-3′ and Bo_GH-R 5′-GAGCTCTTGATGCAATTTCCTCGC-3′. The PCR condition of As_GH-cDNA was denaturation at 94 °C for 1 min, annealing at 60 °C for 2 min, extension at 72 °C for 3 min (35 cycles), and a final extension at 72 °C for 10 min, while Bo_GH-cDNA was denaturation at 94 °C for 1 min, annealing at 62 °C for 2 min, extension at 72 °C for 3 min (35 cycles), and a final extension at 72 °C for 10 min.

### Construction of GH cloning vector

Growth hormone cDNA sequences of Assaf sheep and Boer goat were ligated into pTZ57R/T cloning vector according to the InsTAclone cloning kit procedures (#K1213, Fermentas, USA) as follows: 3 μl of pTZ vector, 3 μl of both As_GH/Bo_GH cDNA, 6 μl of 10× ligase buffer, 1.5 μl T4 DNA ligase, and 16.5 μl water in total 30 μl reaction mixture, then incubated for ligation at 22 °C/1 h. The constructions of As_GH and Bo_GH-PTZ were transformed into DH10B cells for proliferation and extraction. The constructed As_GH and Bo_GH-PTZ vectors were sequenced for GH cDNA sequences. The efficiency of transformation was determined using previous primers and PCR conditions.

### Bioinformatics analysis of As_GH and Bo_GH cDNA sequences

The consensus sequence of As_GH and Bo_GH cDNA was created using the Bioedit 7.2.5 software [8]. The open reading frame (ORF) of As_GH and Bo_GH cDNA sequences was predicted using https://web.expasy.org/translate/ [9]. The cleavage site was predicted using SignalP 4.1 Server tool http://www.cbs.dtu.dk/services/SignalP/ [10]. Predicted As_GH and Bo_GH protein feature annotations have been illustrated using Protter program version 1.0 that available at http://wlab.ethz.ch/protter/# [11].

The cysteine state and disulfide bonds of As_GH and Bo_GH cDNA sequences were carried out using an available tool at http://clavius.bc.edu/~clotelab/DiANNA/ [12]. The physical characteristics of the As_GH and Bo_GH proteins were calculated using the EMBOSS Pepstats tool which is available at https://www.ebi.ac.uk/Tools/seqstats/emboss_pepstats/. The transcription promoters have been predicted using http://www.fruitfly.org/seq_tools/promoter .html [13].

The conserved domains of As_GH and Bo_GH and As_GH cDNA sequences were detected using conserved domains tool http://www.ncbi.nlm.nih.gov/Structure/cdd/wrpsb.cgi [14] The conserved domain multiple alignments were achieved using https://www.st-va.ncbi.nlm.nih.gov/tools/cobalt/cobalt.cgi?CMD=Web. The protein motifs of As_GH and Bo_GH cDNA were predicted using the search motif library tool (https://www.genome.jp/tools/motif/). The protein motifs multiple alignments were achieved using http://meme-suite.org/tools/mast or https://prosite.expasy.org/scanprosite/. Pairwise and multiple alignments for SNPs prediction between As_GH and Bo_GH cDNA sequences and between the GenBank database achieved using the Bioedit 7.2.5 software [8]. The multiple alignments were achieved for As_GH and Bo_GH protein sequences *vs.* GenBank database using the Bioedit 7.2.5 software [8].

## Results

### Growth hormone cDNA isolation and subcloning

**Fig. 1:**
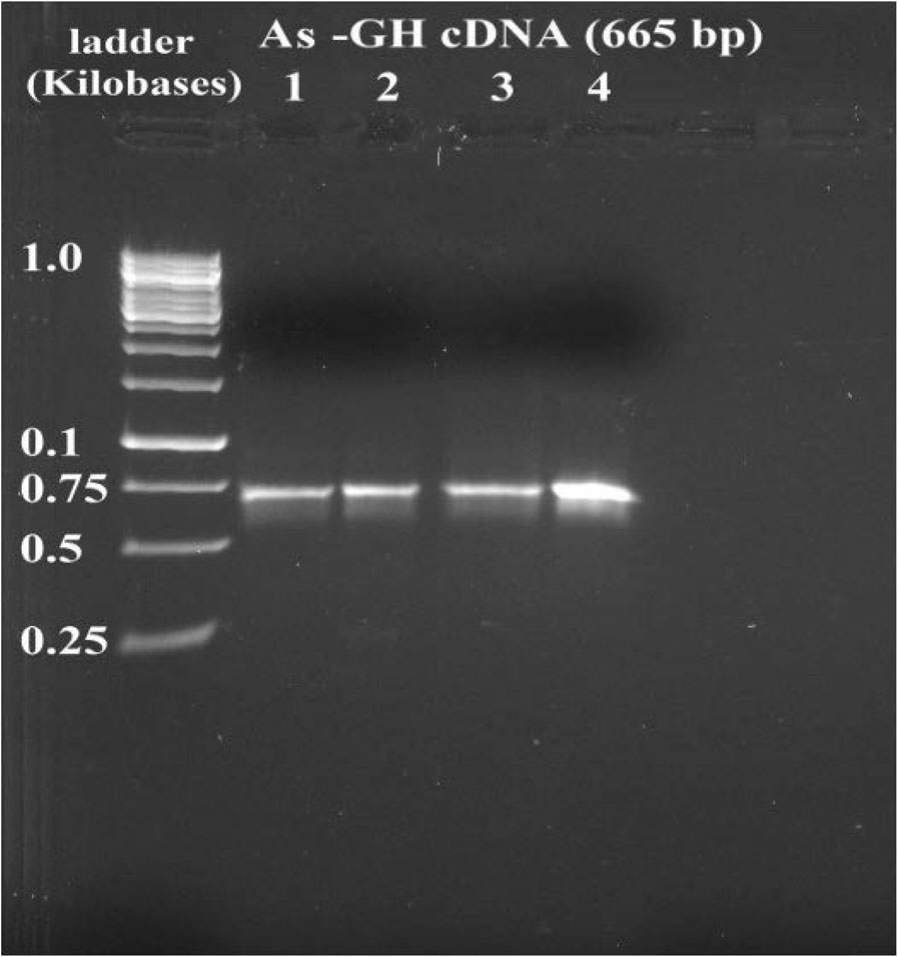
Gel electrophoresis bands of As_GH cDNA

**Fig. 2:**
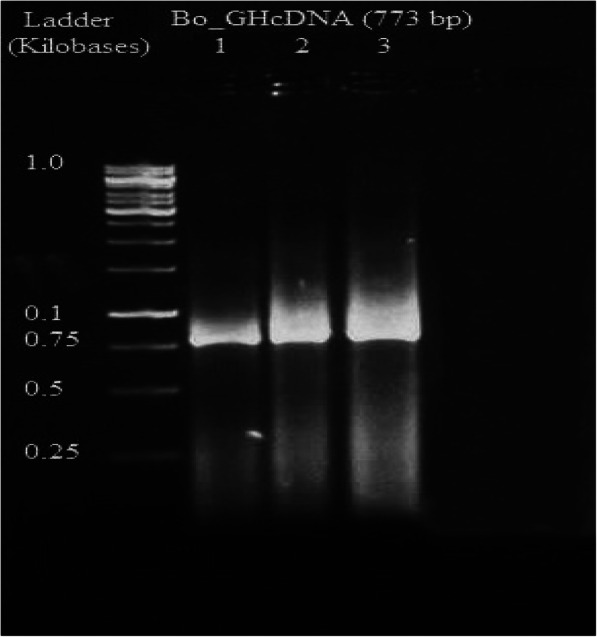
Gel electrophoresis bands of Bo_GH cDNA

**Fig. 3:**
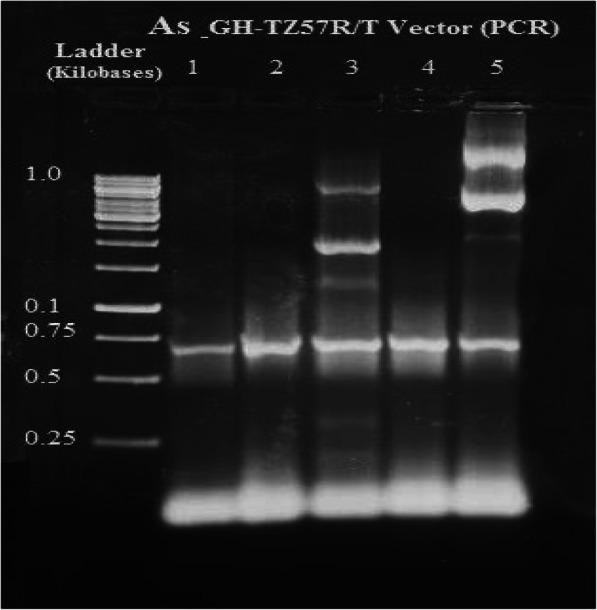
PCR electrophoresis of As_GH-TZ57R/T vector

**Fig. 4:**
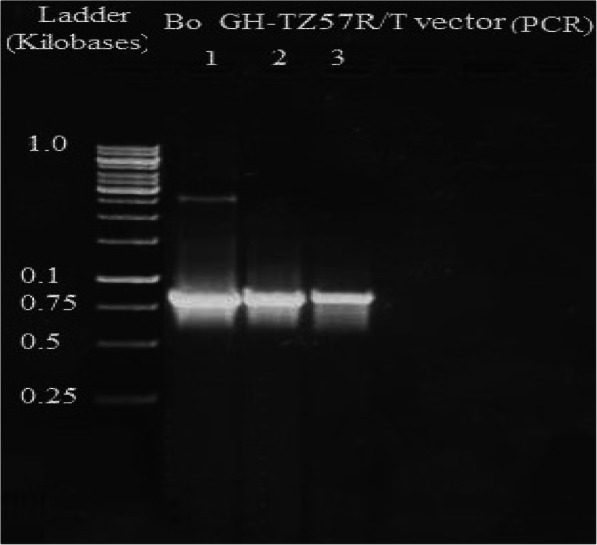
PCR electrophoresis of Bo_GH-TZ57R/T vector

The growth hormone cDNA sequence was isolated from the pituitary gland of Assaf sheep and Boer goat. The molecular weight of the As_GH cDNA sequence was 665 bp and was 774 bp for Bo_GH cDNA (Figs. 1 and 2, respectively). Polymerase chain reaction was used to confirm the successful ligation of As_GH and Bo_GH cDNA sequences into the construction of As_GH-TZ57R/T vector and Bo_GH-TZ57R/T vector (Figs. 3 and 4, respectively). The complete sequences of As_GH and Bo_GH were registered in the GenBank database under accession number MH128986 for the As_GH sequence and MG744290 for the Bo_GH sequence.

### Growth hormone cDNA sequence annotation

#### Assaf and Boar growth hormone SNPs

##### Pairwise alignment of As_GH and Bo_GH cDNA sequence

**Fig. 5:**
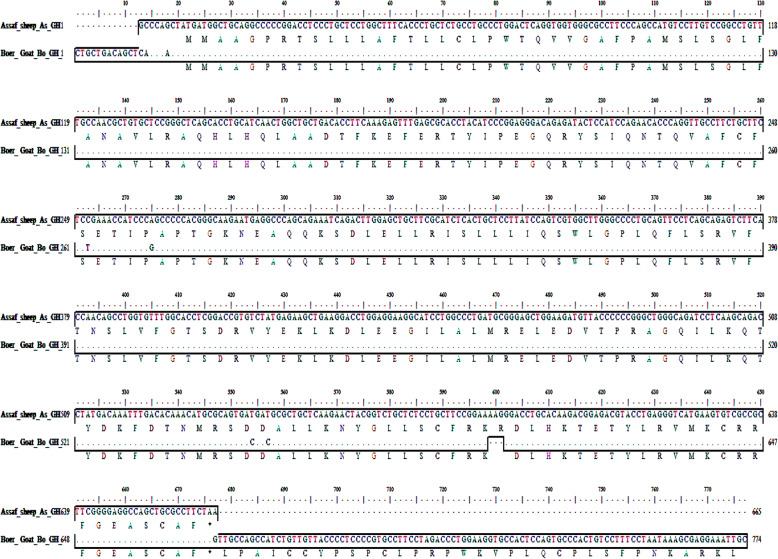
Pairwise alignment of Assaf_GH and Boer_GH cDNA sequences with their predicted protein residues

Pairwise alignment between As_GH and Bo_GH cDNA growth hormone sequences (Fig. 5) indicated that there were four synonymous alleles substitutions (small letter) which yield a new codon that encodes the same amino acids. In Assaf sheep, TCC for serine (Ser, S^251^), CCA for proline (Pro, P^263^), GAT for aspartic acid (Asp, D^542^), and GAT for aspartic acid (Asp, D^545^) versus in Boer goat, TCt for serine (Ser, S^263^), CCg for proline (Pro, P^275^), GAc for aspartic acid (Asp, D^554^), and GAc for aspartic acid (Asp, D^557^), respectively. Assaf sheep has a unique three SNPs (A^637^G^638^G^639^) that encodes for arginine amino acid (Arg, R^194^); this insertion mutation (AAG) has been absent in the growth hormone cDNA sequences of Boer goat in the current study and all breeds in GenBank database.

##### Multiple alignments of As_GH sequence vs. GenBank sheep database

**Fig. 6:**
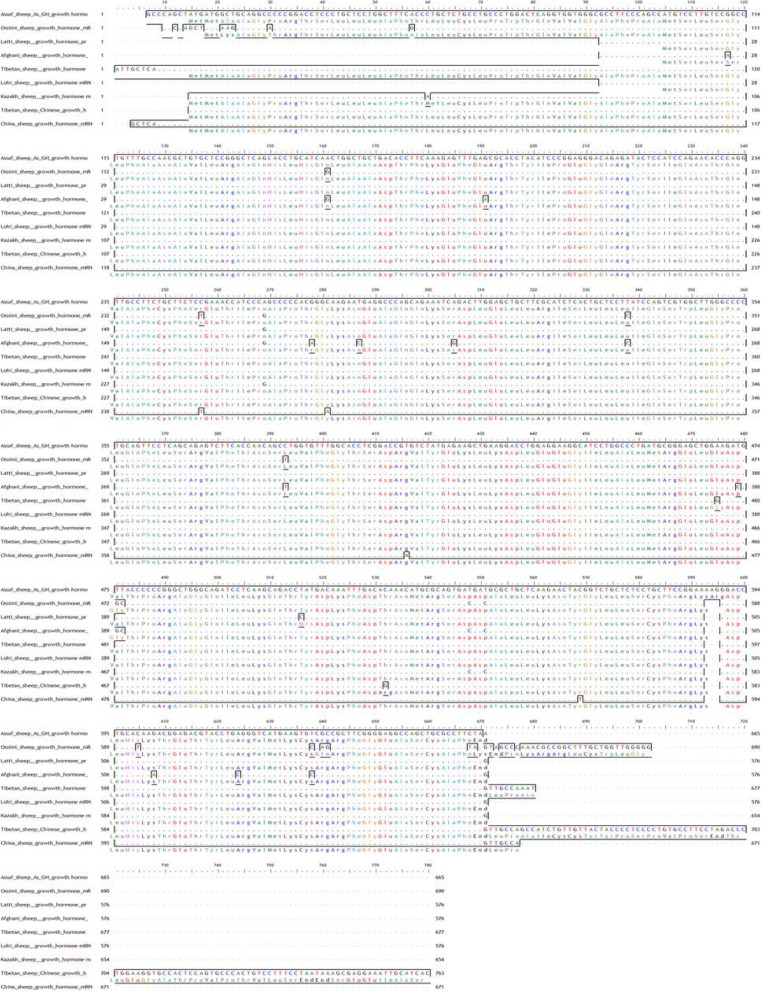
Multiple alignments of Assaf_GH cDNA sequences with GenBank sheep database

Multiple alignments between growth hormone sequence of Assaf sheep and GenBank database (Fig. 6) showed thirty-two synonymous alleles substitutions (small letter) which yield new codons that encode for same amino acids and twelve synonymous alleles substitutions (small letter) which yield new codons that encode for different amino acids (Table 1).

**Table 1:** Multiple alignment of As_GH cDNA sequence with GenBank sheep database

Current Breed	Codon	Residue/position	Codon	Residue/position	GenBank database
**Assaf sheep**	ATGATG	Met^1^Met^2^	ATG	Met^1^	Ossimi sheep (Egypt)
	GCT	Ala^3^	a^12^a^13^g^14^	Lys^2^	
	C^24^CC	Pro^6^	a^21^CC	Thr^5^	
	A^51^CC	Thr^15^	g^48^CC	Ala^14^	
	CAA^155^	Gln^75^	CAg^152^	Gln^74^	
	TCC^251^	Ser^81^	TCt^248^	Ser^80^	
	CCA^363^	Pro^85^	CCg^260^	Pro^84^	
	CTT^332^	Leu^108^	CTc^329^	Leu^107^	
	C^387^TG	Leu^127^	T^384^TG	Leu^126^	
	GT^475^T^476^	Val^156^	Gg^472^c^473^	Gly^155^	
	GAT^542^	Asp^178^	GAc^539^	Asp^177^	
	GA^545^G	Asp^179^	Gc^542^G	Asp^178^	
	CAC^599^	His^197^	CAt^593^	His^195^	
	TGT^632^	Cys^208^	TGc^626^	Cys^205^	
	CG^634^C^635^	Arg^209^	Ca^628^g^629^	Gln^207^	
	TTC^662^	Phe^218^	TTt^656^	Phe^216^	
	T^663^AA	Stop codon^219^	a^657^AA	Lys^217^	
	G^111^GC	Gly^35^	A^25^GC	Ser^5^	Afghani breed (Pakistan)
	CAA^161^	Gln^49^	Cag^69^	Gln^19^	
	GAG^191^	Glu^59^	GAa^99^	Glu^29^	
	CCA^269^	Pro^85^	CCg^177^	Pro^55^	
	ACG^278^	Thr^88^	Aca^186^	Thr^186^	
	AAT^287^	Asn^91^	Aac^195^	Asn^61^	
	TCA^305^	Ser^97^	TCg^213^	Ser^67^	
	CTT^338^	Leu ^108^	CTc^246^	Leu^77^	
	CTG^393^	Leu^127^	T^301^TG	Leu^97^	
	GAT^479^	Asp^155^	GAc^390^	Asp^125^	
	GT^481^T^482^	Val^156^	Gg^389^c^390^	Gly^126^	
	GAT^548^	Asp^178^	GAc^456^	Asp^148^	
	GAT^551^	Asp^179^	GAc^459^	Asp^149^	
	AAG^608^	Lys^198^	AAa^513^	Lys^167^	
	A^624^GG	Arg^204^	C^529^GG	Arg^173^	
	TGT^638^	Cys^208^	TGc^543^	Cys^177^	
	C^54^TG	Leu^16^	a^46^TG	Met^16^	Kazakh sheep (China)
	CCa^263^	Pro^85^	CCg^255^	Pro^85^	
	GAT^542^	Asp^178^	GAc^534^	Asp^178^	
	GAT^545^	Asp^179^	GAc^537^	Asp^179^	
	CCa^263^	Pro^85^	CCg^177^	Pro^55^	Latti sheep (Pakistan)
	T^510^AT	Tyr^168^	C^424^AT	His^138^	
	GA^469^A	Glu^154^	Gg^475^A	Gly^154^	Tibetan sheep (China)
	AC^526^A	Thr^173^	Ag^518^A	Arg^173^	
	TCC^251^	Ser^81^	TCt^254^	Ser^81^	China sheep
	GGC^89^	Gly^89^	GGa^278^	Gly^89^	
	CGT^416^	Arg^134^	CGa^413^	Arg^134^	
	TAC^563^	Tyr^185^	Tat^566^	Tyr^185^	
	A^637^G ^638^G^639^	Arg^194^	A distinct mutation, it is absent in sheep or goat breeds		

Also, the unique three SNPs (A^637^G^638^G^639^) of the Assaf growth hormone have been confirmed in the result of the multiple alignments comparing with of all breeds in the GenBank database. Growth hormone cDNA sequences of Ossimi sheep have one start codon (ATG) and three unique SNPs (A^12^A^13^G^14^) encodes for lysine (Lys^2^, K) *vs.* Alanine (Ala^3^, A) in all GenBank database. There are two alleles substitutions in Ossimi and Afghani sheep which yield a new codon that encodes for different amino acid; for Ossimi sheep, Gg^472^c^473^ encodes for glycine (Gly, G), and Ca^628^g^629^ encodes for glutamine (Gln, Q) for Afghani sheep, respectively compared to GTT for valine (Val, V) and CGC for arginine (Arg, R) in the GenBank database and in Afghani sheep, Gg^389^c^390^ for glycine (Gly, G) compared to GTT for valine (Val, V) in the GenBank database.

##### Multiple alignments of Bo_GH sequence vs. GenBank goat database

**Fig. 7:**
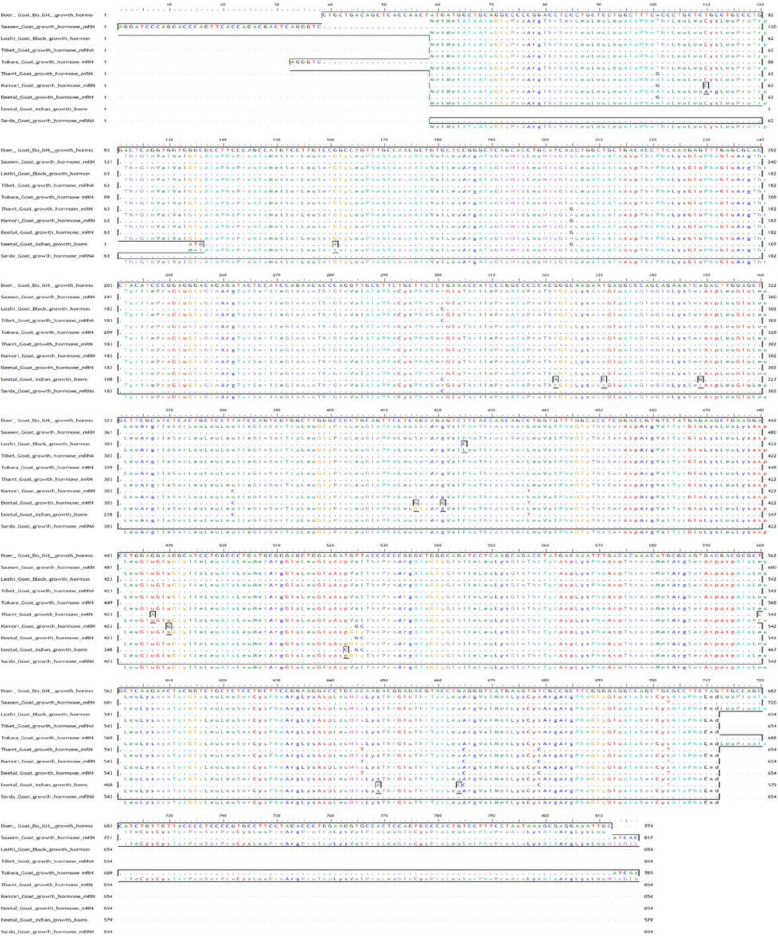
Multiple alignments of Boer_GH cDNA sequences with GenBank goat database

Multiple alignments between growth hormone sequence of Boer goat and GenBank goat database (Fig. 7) showed nine synonymous alleles substitutions (small letter) which yield for new codons that encode for same amino acids: Gln, Ser, Thr, Asn, Cys, Leu, Arg, Glu, and His residues (Table 2). On the other hand, there are six synonymous alleles substitutions (small letter) which yield for new codons that encode for different amino acids: Ala to Thr, Pro to Leu, Gly to Val, Gly to Ser, Phe to Leu, and Arg to Cys. The Bo_GH cDNA sequence has no SNPs compared to the growth hormone sequence at GenBank goat database (Table 2).

**Table 2:** Multiple alignment of Bo_GH cDNA sequence with GenBank goat database

Current breed	Codon	Residue/position	Codon	Residue/position	GenBank database
**Boer goat**	A^63^CC	Thr^15^	g^43^CC	Ala^15^	Tharri goat (Pakistan)
					Kamori goat (Pakistan)
					Beetal goat (Pakistan)
	T^72^GC	Cys^18^	c^52^GC	Arg^18^	Kamori goat (Pakistan)
	G^96^G^97^C^98^	Gly^26^	a^1^t^2^g^3^	Met1	Beetal goat (Indian)
	G^123^GC	Gly^35^	a^28^GC	Ser^10^	Beetal goat (Indian)
	CAA^167^	Gln^49^	CAg^147^	Gln^49^	Tharri goat (Pakistan)
					Kamori goat (Pakistan)
					Beetal goat (Indian)
			CAg^72^	Gln^24^	Beetal goat (Pakistan)
	TCT^263^	Ser^81^	TCc^243^	Ser^81^	Lezhi Black goat (China)
					Tibet goat
					Sarda goat
			TCc^168^		Beetal goat (Indian)
	ACG^284^	Thr^63^	ACa^189^	Thr^88^	Beetal goat (Indian)
	AAT^293^	Asn^91^	AAc^198^	Asn^67^	
	TCA^311^	Ser^97^	TCg^216^	Ser^71^	
	CTT^344^	Leu^108^	CTc^324^	Leu^108^	Kamori goat (Pakistan)
					Beetal goat (Pakistan)
			CTc^249^	Leu^83^	Beetal goat (Indian)
	A^378^GC	Ser^120^	g^358^GC	Gly^120^	Beetal goat (Pakistan)
	AGA^378^	Arg^121^	AGg^363^	Arg^121^	
	T^387^TC	Phe^123^	c^367^TC	Leu^123^	Lezhi Black goat (China)
	C^399^TG	Leu^127^	t^379^TG	Leu^128^	Kamori goat (Pakistan)
					Beetal goat (Indian)
			t^304^TG	Leu^103^	Beetal goat (Pakistan)
	GAG^449^	Glu^143^	GAa^429^	Glu^143^	Tharri goat (Pakistan)
	GAA144	Glu^144^	Gag^432^	Glu^144^	Kamori goat (Pakistan)
	GT^487^T^488^	Val^156^	Gg^467^c^468^	Gly^156^	Kamori goat (Pakistan)
					Beetal goat (Indian)
			Gg^392^c^393^	Gly^131^	Beetal goat (Pakistan)
	CT^562^G	Leu^181^	Cc^542^G	Pro^181^	Tharri goat (Pakistan)
	CAC^608^	His^169^	CAt^588^	His^197^	Tharri goat (Pakistan)
					Kamori goat (Pakistan)
					Beetal goat (Pakistan)
	A^627^GG	Arg^203^	c^607^GG	Arg^203^	Tharri goat (Pakistan)
					Kamori goat (Pakistan)
					Beetal goat (Pakistan)
			c^532^GG	Arg^179^	Beetal goat (Indian)
	TGT^665^	Cys^207^	TGc^207^	Cys^207^	Tharri goat (Pakistan)
					Kamori goat (Pakistan)
					Beetal goat (Pakistan)
			TGc^182^	Cys^182^	Beetal goat (Indian)
	TGC^665^	Cys^215^	TGt^645^	Cys^215^	Tharri goat (Pakistan)
					Kamori goat (Pakistan)
					Beetal goat (Pakistan)
			TGt^570^	Cys^190^	Beetal goat (Indian)

Pakistani Kamori, Tharri, and Beetal goat shared with the same SNPs encode for the same amino acids: g^43^CC for Ala^15^, CAt^588^ for His^197^, c^607^GG for Arg^203^, TGc^207^for Cys^207^, and TGt^645^ for Cys^215^ vs. A^63^CC for Thr^15^, CAC^608^ for His^169^, A^627^GG for Arg^203^, TGT^665^ for Cys^207^, and TGC^665^ for Cys^215^ compared to the GenBank goat database, respectively.

Single nucleotide polymorphisms of A/G was observed in Afghani sheep (a^25^GC that encoded for Ser^5^) and Indian Beetal goat (a^28^GC that encoded for Ser^10^) vs*.* G^111^GC that encoded for Gly^35^ in GenBank sheep database and GGC in GenBank goat database, respectively. In Pakistani Beetal goat has G/A SNP, g^358^GC (Gly^120^) compared to A^78^GC (Ser^120^) in GenBank goat database.

### The prediction of growth hormone promoter

**Fig. 8:**
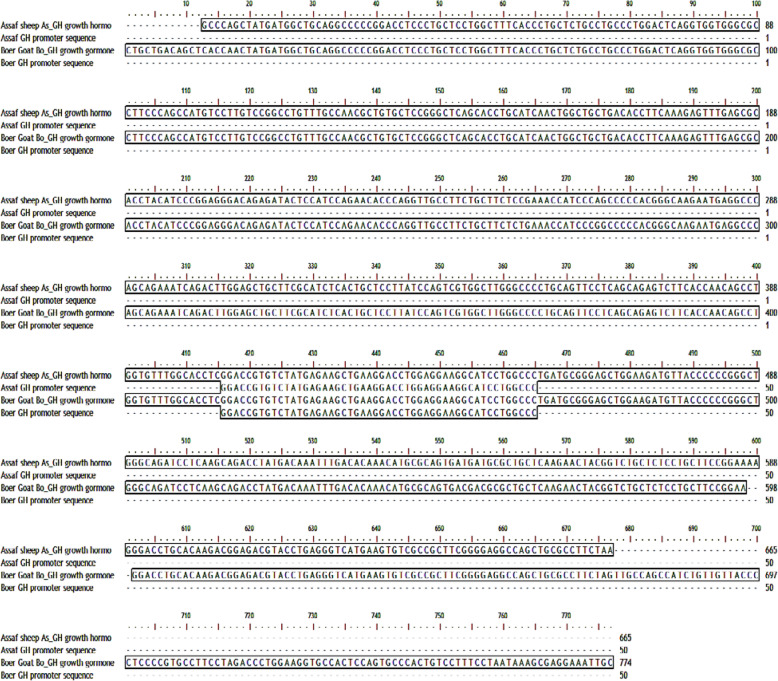
Pairwise alignment of As_GH and Bo_GH cDNA sequences and their promoter sequence

The As_GH and Bo_GH cDNA sequences and the predicted promoters were shown in Fig. 8. The As_GH promoter sequence was spanned from 404 to 454 bp (50 bp length) with a 0.74 prediction score. The sequence included the ATG start codon CCTCGGACCGTGTCT**ATG**AGAAGCTGAAGGACCTGGAGGAAGGCATCCT. While the promoter sequence of the Bo_GH cDNA sequence was GGACCGTGTCT**ATG**AGAAGCTGAAGGACCTGGAGGAAGGCATCCTGGCCC (50 bp length), contained start codon (ATG) and spanned from 416 to 466 bp with prediction score 0.74. The identity score between both promoters was 100% irrespective of position difference. The promoter sequence in As_GH and Bo_GH sequence was completely identical and has the same length irrespective of position difference.

### Protein structure annotation

#### Signal peptides sequence

**Fig. 9:**
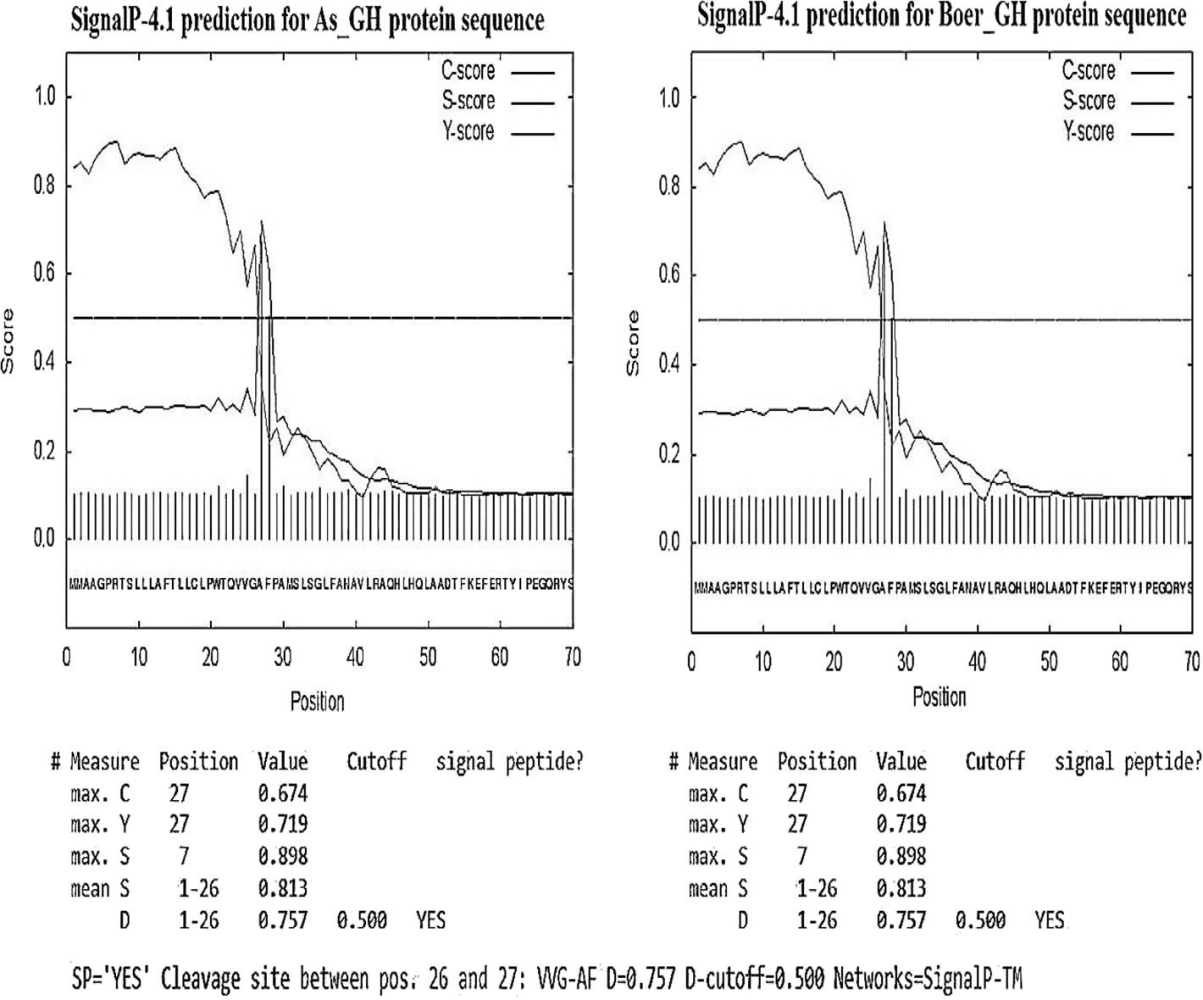
The predicted cleavage site of signal peptide in As_GH and Bo_GH protein sequences

The signal peptides ranged from 1 to 27 residues for both As_GH and Bo_GH protein sequences with 100% similarity sequence peptides. The predicted cleavage site was located between 26 and 27 amino acids in both Bo_GH and As_GH protein (Fig. 9).

#### Conserved domain and motifs

**Fig. 10:**
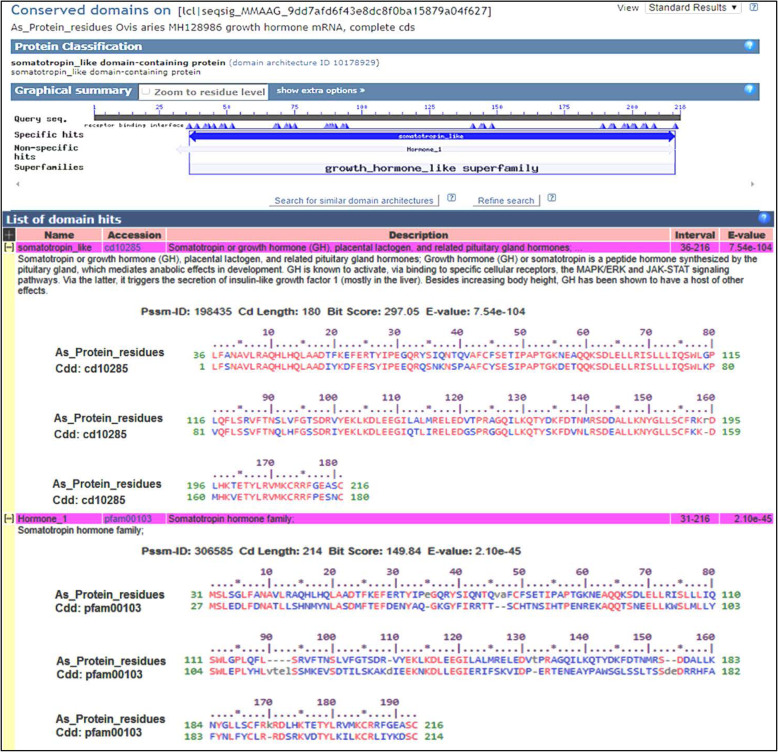
Conserved domain of As_GH protein residues

**Fig. 11:**
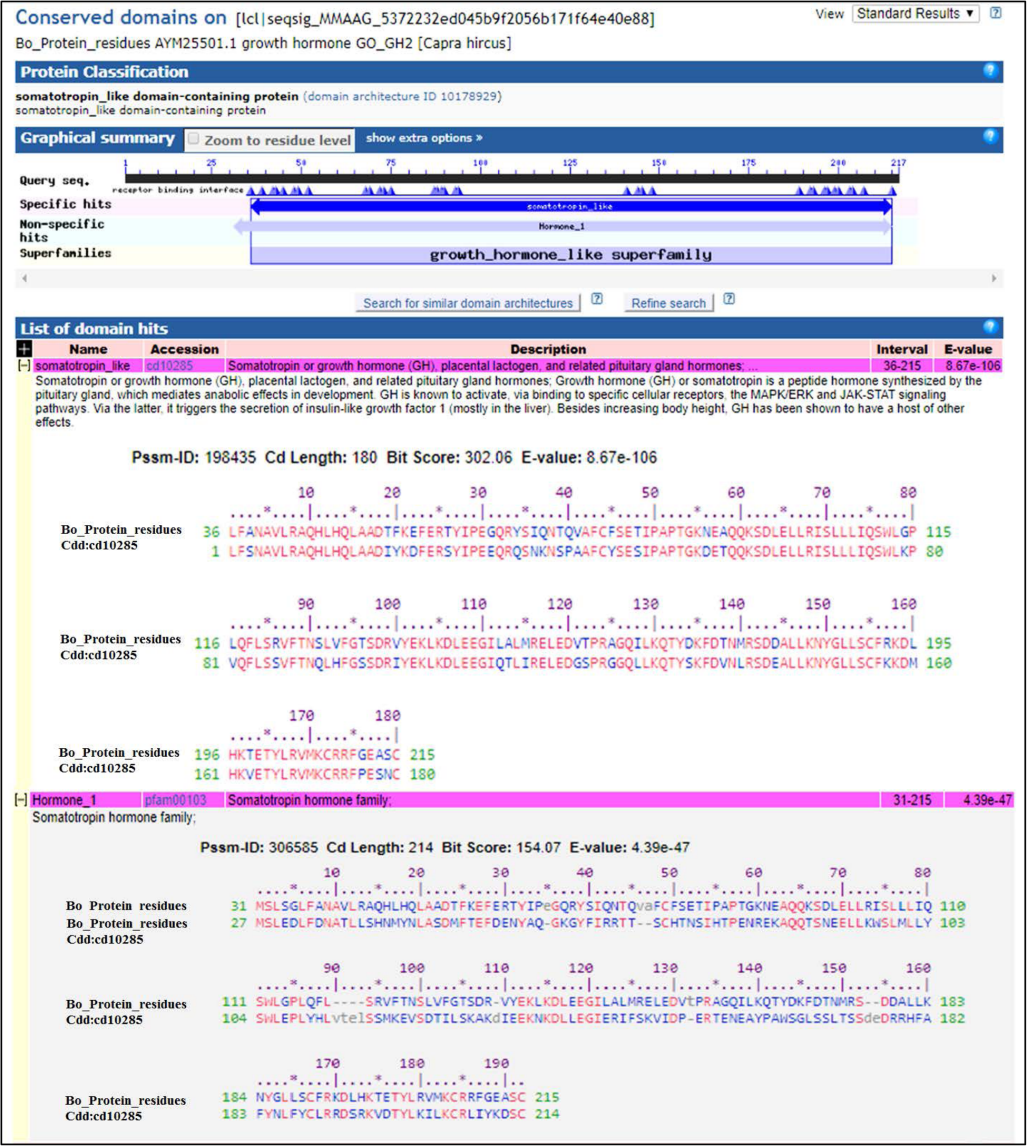
Conserved domain of Bo_GH protein residues

**Fig. 12:**
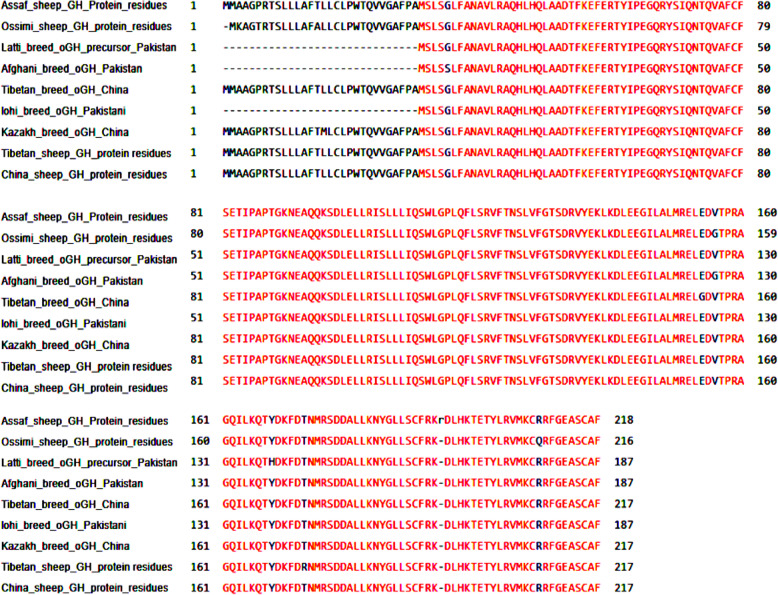
Multiple alignment of conserved domain of As_GH protein residues with GenBank database

The conserved domain of As_GH sequence protein was matched with two hits, growth hormone-like superfamily that interval from 31 to 216 aa and somatotropin like that interval 36–216 aa. Also, Bo_GH sequence protein has two hits, growth hormone-like superfamily that interval from 31 to 215 aa and somatotropin like that interval from 36–215 aa (Figs. 10 and 11, respectively). The multiple alignments of conserved domain of the As_GH protein sequence and GenBank sheep database (Fig. 12) showed that the conserved domain of all breeds was matched with growth hormone-like superfamily hit that started from protein methionine (Met^31^), and there were six different substitution amino acids in four breeds: Ser^34^ and Gly^125^ in Afghani, Gly^155^ and Gln^206^ in Ossimi, Gly^153^ in Tibetan, and His^138^ and Arg^173^ in Latti sheep compared to Gly, Val, Val, Arg, Glu, Tyr, and Thr, respectively in the GenBank sheep breeds. The As_GH sequence protein has a unique amino acid Arg^194^, and it is absent in all GenBank sheep database.

**Fig. 13:**
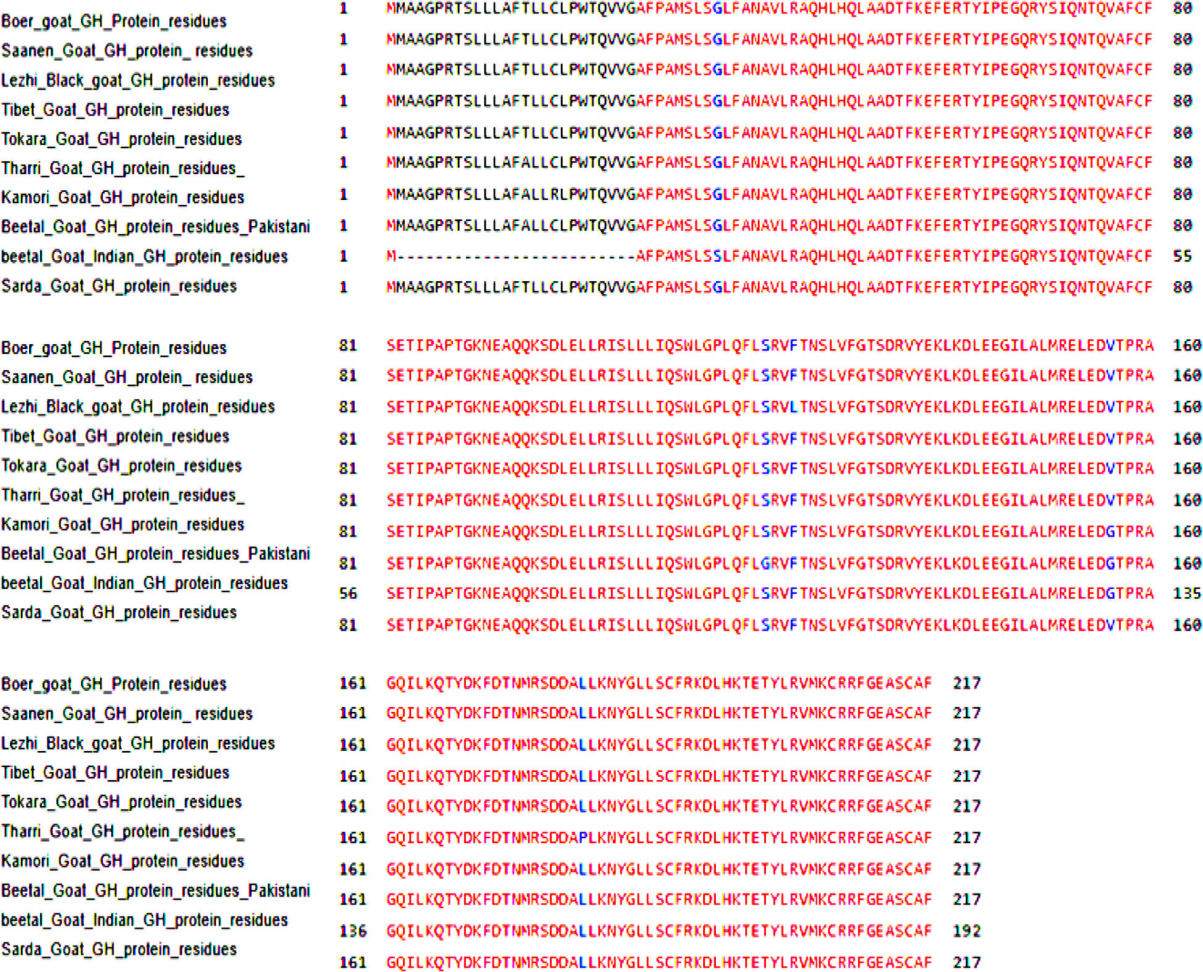
Multiple alignment of conserved domain of Bo_GH protein residues with GenBank database

The multiple alignments of the conserved domain of the Boer goat and GenBank goat database (Fig. 13) showed that the conserved domain of all breeds was shared with somatolactin (SL) and somatolactin-like protein hit that ranged from Ala^27^ and included the previous two hits growth hormone-like superfamily and somatotropin like hit. There were six different substitution amino acids in five breeds: Gly^120, 156^ in Pakistani Beetal, Leu^123^ in Lezhi, Gly^156^ in Kamori, Ser^10^ and Gly^131^ in Indian Beetal, and Pro^181^ in Thari goat compared to Ser, Val, Phe, Val, Gly, and Val in the GenBank sheep breeds, respectively. There was no different substitution in the Bo_GH protein sequence compared to the GenBank goat database.

**Fig. 14:**
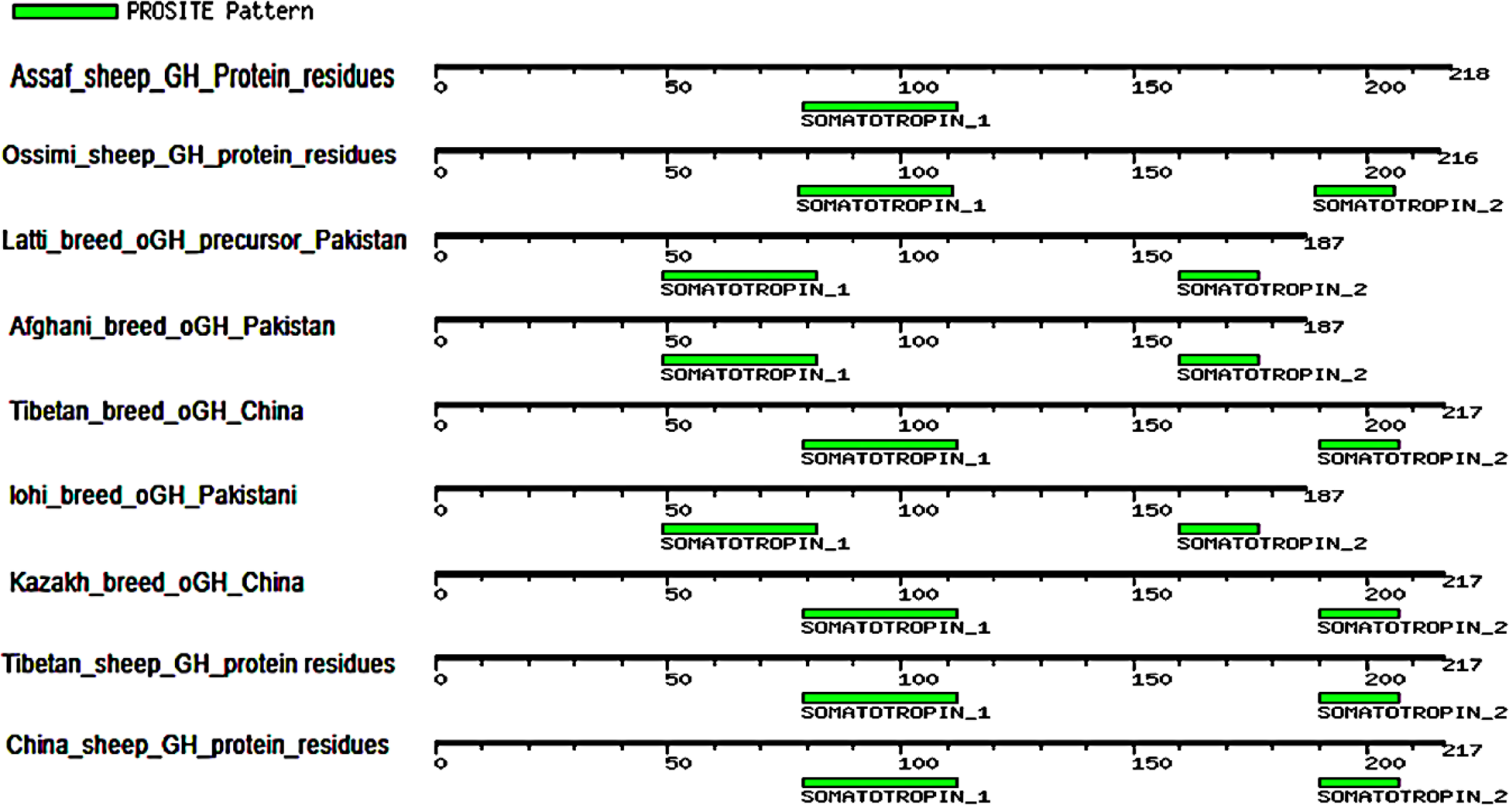
Motif patterns of As_GH protein sequence compared with GenBank database

**Fig. 15:**
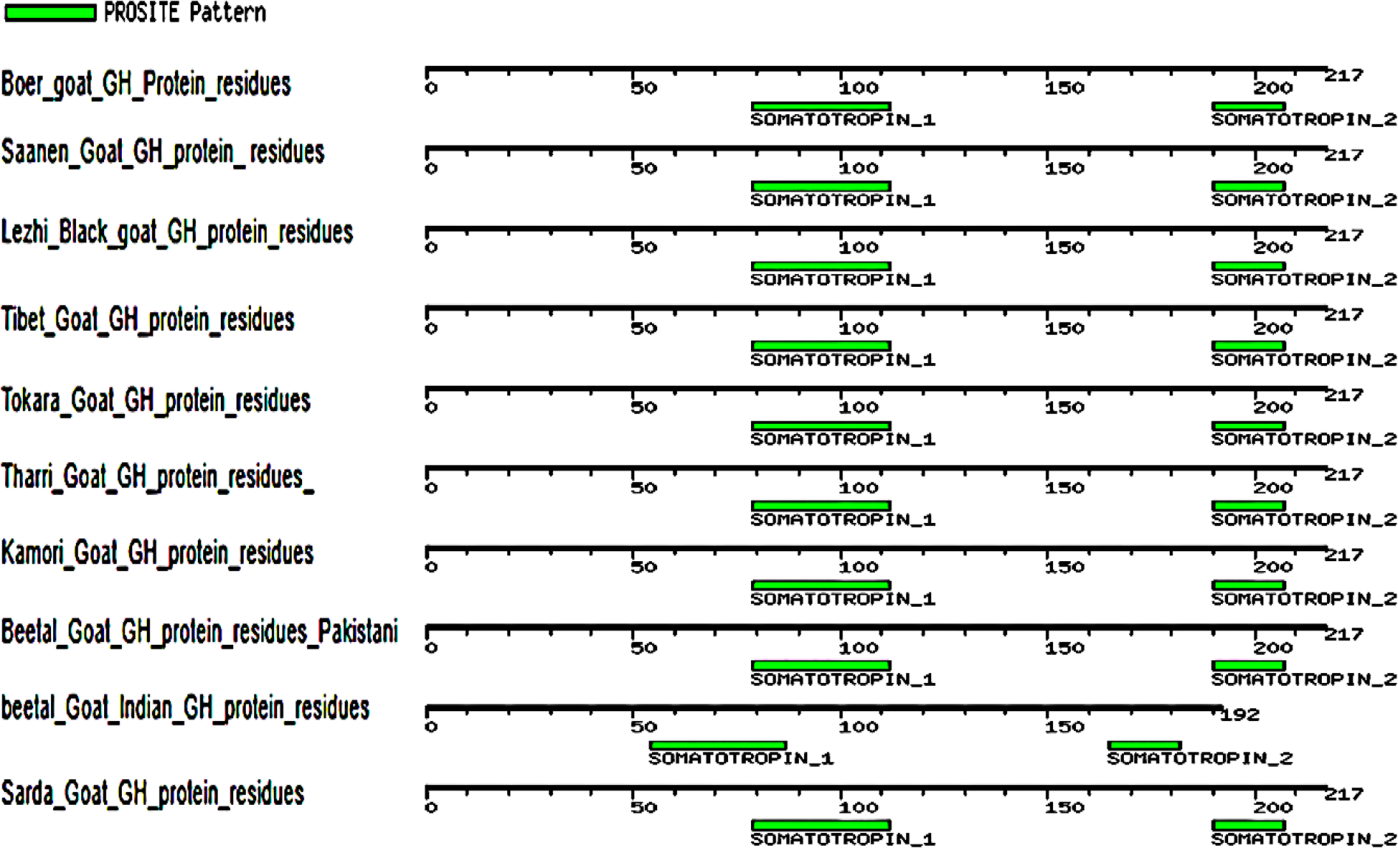
Motif patterns of Bo_GH protein sequence compared with GenBank database

The multiple alignments of the As_GH protein sequence with the GenBank database showed that the As_GH protein sequence has one motif signature, Somatotropin_1 (CFSETIPAPT GKNEAQQKSDLELLRISLLLIQSW) from 79 to 112 aa (Fig. 14), while the multiple alignments of the Bo_GH protein sequence with the GenBank database showed two motifs, Somatotropin_1 (191 related sequences) and Somatotropin_2 (195 related sequences). In the present study, the Bo_GH sequence protein had two common motifs signature (Fig. 15): Somatotropin_1 (CFSETIPAPTGKNEAQQKSDLELLRISLLLIQSW) from 79 to 112 aa and Somatotropin_2 (CFRKDLHKTETYLRVMKC) from 190 to 207 aa.

#### Cysteine bridge and disulfide bonds

**Fig. 16:**
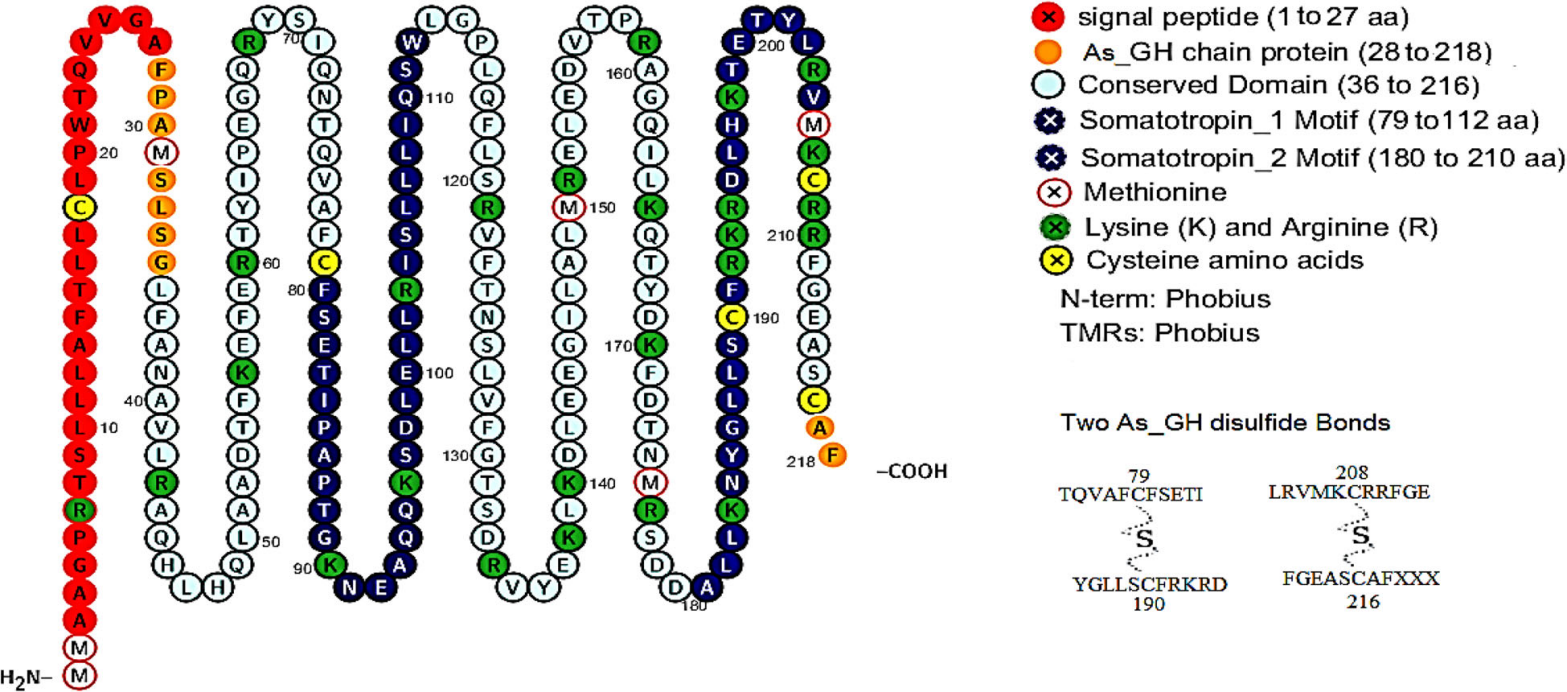
The Assaf sheep GH_cDNA protein features and disulfide bounds

**Fig. 17:**
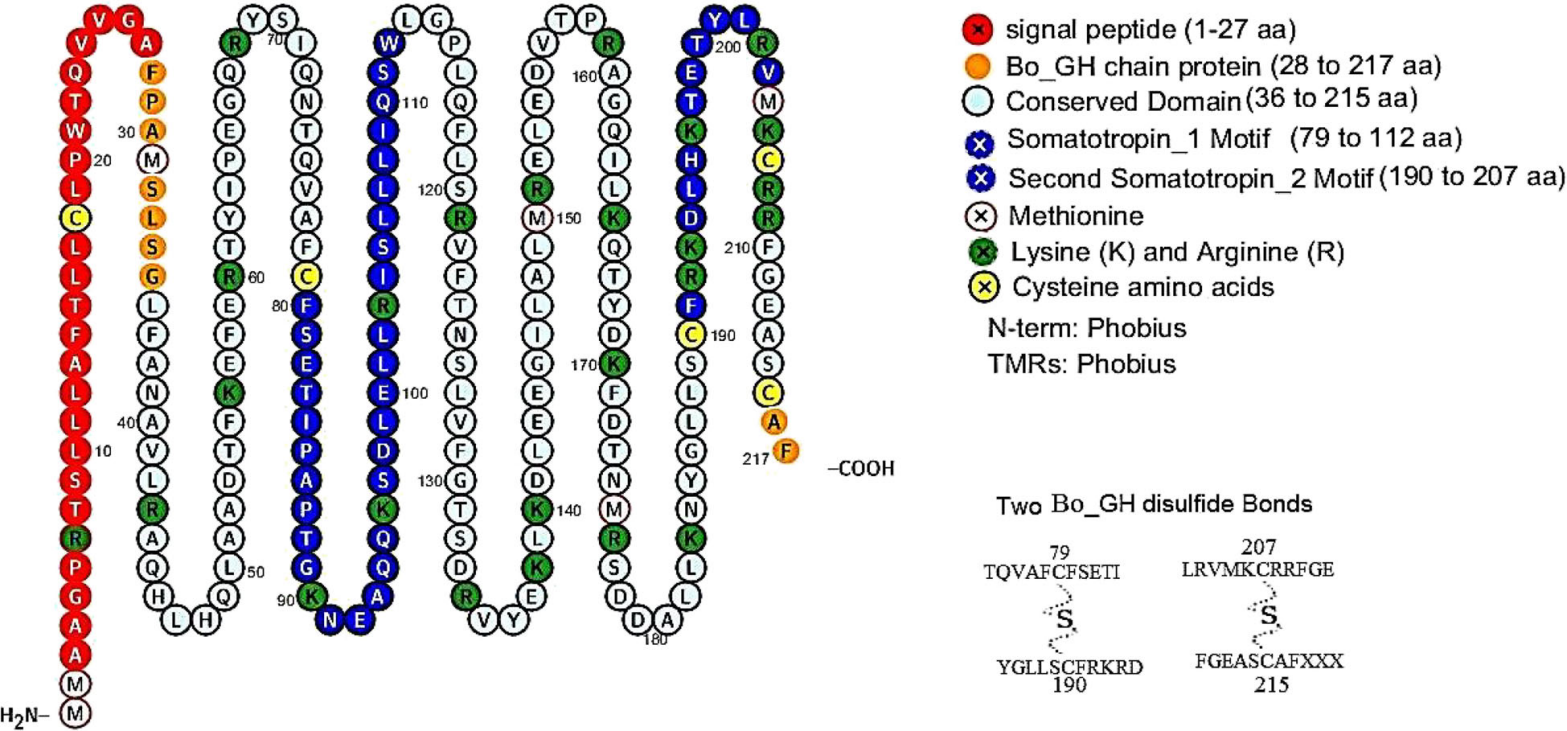
The Boer goat GH_cDNA protein features and disulfide bounds

Five conserved cysteines (Cys) residues were detected in both As_GH and Bo_GH protein sequences at the following positions: Cys^18^, Cys^79^, Cys^190^, Cys^208^, and Cys^216^ in As_GH protein sequence and at Cys^18^, Cys^79^, Cys^190^, Cys^207^, and Cys^215^ in Bo_GH protein sequence. Two sulfide bonds were predicted between Cys^79^, Cys^190^ and between Cys^208^ and Cys^216^ aa in As_GH protein and between Cys^79^, Cys^190^ and between Cys^207^ and Cys^215^ in Bo_GH protein (Figs. 16 and 17, respectively). The comprehensive summary annotation of As_GH and Bo_GH protein residues was shown in Table 3.

**Table 3:** Comprehensive summary of As_GH and Bo_GH annotations

Parameters	As_GH cDNA sequence			Bo_GH cDNA sequence		
**Length of mRNA**	665 bp			774 bp		
**Predicted promoter**	Start 404, end 454, score 0.74			Start 416, end 466, score 0.74		
**Physical characteristics of growth hormone protein**						
**Length of protein**	218 aa			217 aa		
**Molecular weight**	24786.7 bp			24630.5 bp		
**Chain peptide**	28–218 (190 aa)			28–217 (189 aa)		
**Open reading frame (ORF)**	1–218			1–217		
**Signal peptide**	1–27			1–27		
**Conserved domain**	36–216			36–215		
**Protein properties**	**Residues**	**No.**	**Mole %**	**Residues**	**No.**	**Mole %**
**Tiny**	A,C,G,S,T	63	28.9	A,C,G,S,T	63	29.0
**Small**	A,B,C,D,G,N,P,S,T,V	96	44.0	A,B,C,D,G,N,P,S,T,V	96	44.2
**Aliphatic**	A,I,L,V	67	30.7	A,I,L,V	67	30.9
**Aromatic**	F,H,W,Y	25	11.5	F,H,W,Y	25z	11.5
**Non-polar**	A,C,F,G,I,L,M,P,V,W,Y	119	54.6	A,C,F,G,I,L,M,P,V,W,Y	119	54.8
**Polar**	D,E,H,K,N,Q,R,S,T,Z	99	45.4	D,E,H,K,N,Q,R,S,T,Z	98	45.2
**Charged**	B,D,E,H,K,R,Z	52	23.9	B,D,E,H,K,R,Z	51	23.5
**Basic**	H,K,R	29	13.3	H,K,R	28	12.9
**Acidic**	B,D,E,Z	23				10.6
**Motifs**	**Position**			**Position**		
**Somatotropin_1**	79–112			79–112		
**Somatotropin_2**	–			190–207		
**Disulfide bonds**	79–190			79–190		
	208–216			207–215		
**Cysteine position**	18			18		
	79			79		
	190			190		
	208			207		
	216			215		

### Protein alignments of As_GH and Bo_GH predicted protein sequence

#### Pairwise alignments

**Fig. 18:**
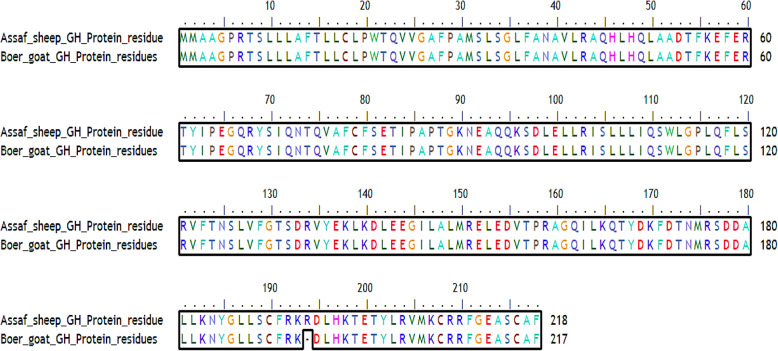
Pairwise alignments of As_GH and Bo_GH protein sequences

The pairwise alignments of As_GH and Bo_GH predicted proteins (Fig. 18) indicated that the homology percentage of growth hormone amino acids in both breeds reached 99.5%. As_GH protein sequence has a unique residue (arginine, R^194^) that was absented in the Bo_GH protein sequence.

#### Multiple alignments of growth hormone protein residues

**Fig. 19:**
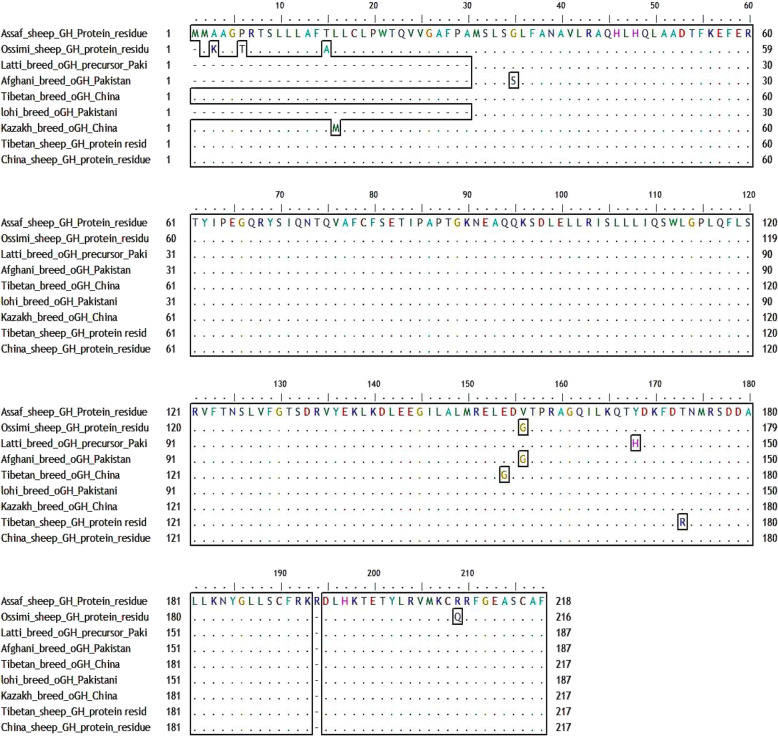
Multiple alignment of As_GH protein sequence vs. GH protein sequence at GenBank sheep database

The multiple alignments of As_GH protein sequences *vs.* GenBank protein database (Fig. 19) showed eleven modified residues in growth hormone protein sequence in five sheep breeds: Assaf, Ossimi, Afghani, Latti, and Tibetan. The unique residue of Arg^194^ in As_GH protein sequence was absented in all growth hormone protein sequences of sheep and goat breeds at the GenBank database. All breeds in the present study and GenBank database started with two start codons (MM) except Egyptian Ossimi sheep that have one start codon. Ossimi sheep have five modified amino acids’ substitution: Lys^2^, Thr^5^, Ala^14^ (signal peptide), Gly^155^, and Gln^207^ vs. Ala^3^, Pro^6^, Thr^15^ (signal peptide), Val^156^, and Arg^209^ in the GenBank database, respectively. In Afghani and Tibetan sheep have two modifies amino acids substitution: Ser^5^, Gly^126^ and Gly^154^, Arg^175^ vs*.* Gly^35^, Val^156^ and Glu^154^, Thr^176^, respectively compared to the GenBank database. The sheep GenBank database has dominant SNP at AC^526^A that encoded for Thr^173^ compared to Ag^518^A encoded for Arg^173^ in Chines Tibetan sheep.

**Fig. 20:**
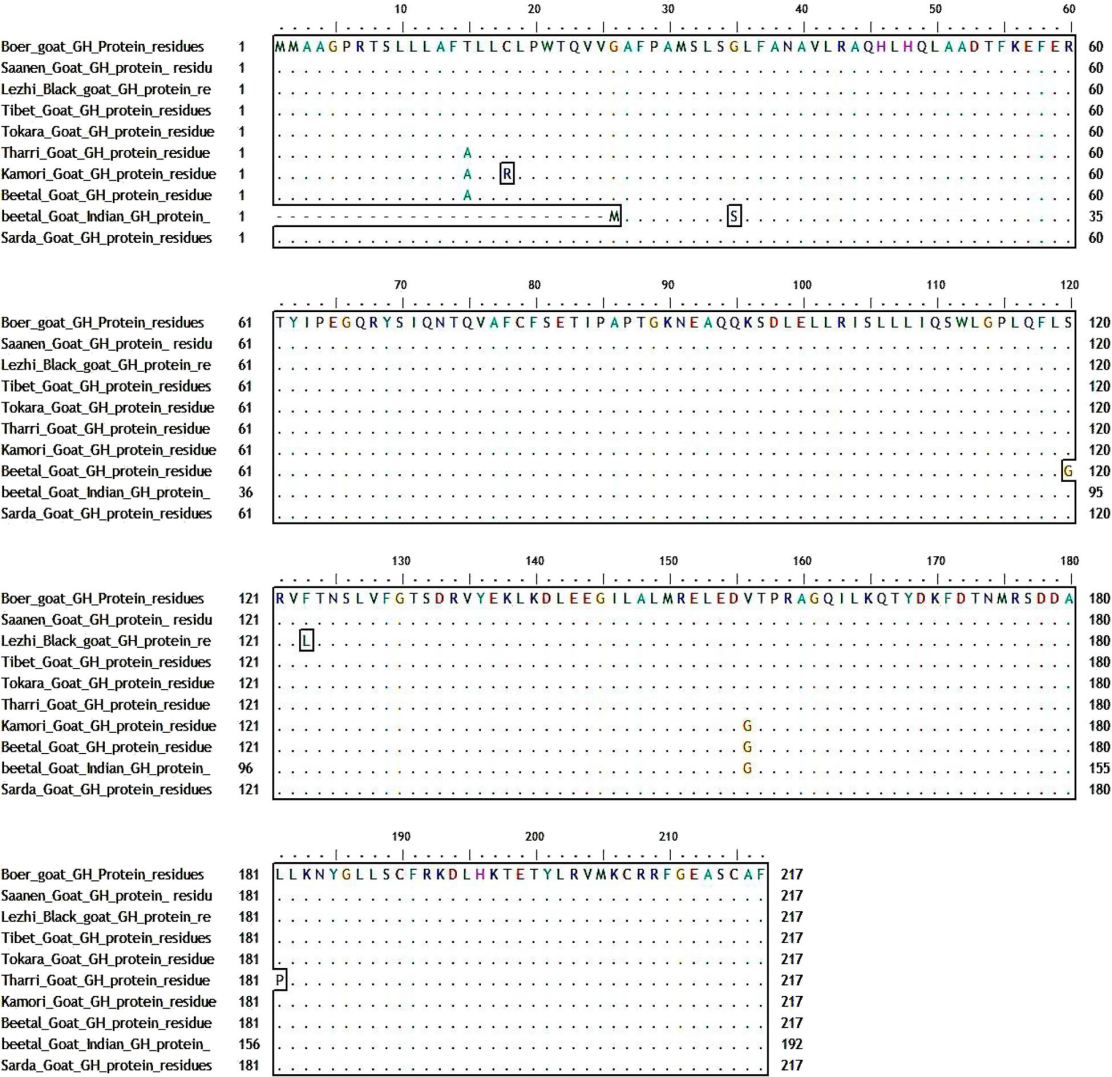
Multiple alignment of Bo_GH protein sequence vs*.* GH protein sequence at GenBank goat database

The multiple alignments of Bo_GH protein sequences vs. GenBank growth hormone protein database (Fig. 20) showed twelve modified amino acids in five goat breeds: Tharri, Kamori, Pakistani Beetal, India Beetal, and Lezhi. Pakistani goat breeds (Tharri, Kamori, and Beetal) were shared in the same amino acid substitution of alanine at the same position Ala^15^ vs. Threonine (Thr^15^), respectively in Bo_GH and GenBank growth hormone protein sequences of goat breeds. The Pakistani Kamori and Beetal goat breed have the same amino acid substitution of glycine at the same position (Gly^156^), while, was at a different position (Gly^131^) in Indian Beetal goat vs. valine (Val^156^), respectively compared to growth hormone protein sequence of Boer and GenBank goat breeds. The multiple alignments showed that Kamori goat has Arg^18^ substitution vs. Cys^18^; Indian Beetal has Met^1^ and Ser^10^ substitution vs. Gly^26^ and Gly^35^; Pakistani Beetal has Gly^120^ substitution vs*.* Ser^120^; Tharri goat has Pro^181^ substitution vs. Leu^181^; Lazhi goat has Leu^123^ substitution vs. Pro^123^ compared to GenBank database.

## Discussion

### Growth hormone cDNA subcloning in TZ57R/T vector

The electrophoretic pattern of As_GH-TZ57R/T vector and Bo_GH-TZ57R/T constructions were confirmed the successful ligation of As_GH and Bo_GH cDNA sequences into the construction of TZ57R/T cloning vector (Figs. 3 and 4, respectively).

### Growth hormone cDNA sequence annotation

#### Assaf and Boar growth hormone SNPs

Single nucleotide polymorphisms (SNPs) in different candidate genes and their association with animal performance have investigated in different animal species: cattle [15–17], sheep [18, 19], and goats [20]. The growth hormone gene SNPs have widely used for the genetic marker as an aid to genetic selection in farm animals: cattle [17, 21–23], sheep [24], and goat [25, 26].

#### Pairwise alignment of As_GH and Bo_GH cDNA sequence

Assaf sheep has a unique three SNPs (A^637^G^638^G^639^) that encodes for arginine amino acid (Arg, R^194^); this insertion mutation (AAG) has been absent in the growth hormone cDNA sequences of Boer goat in the current study and all breeds in GenBank database. This SNPs (AAG) may be used to develop a genetic marker for Assaf sheep breed.

#### Multiple alignments of As_GH sequence vs. GenBank sheep database

Also, the unique three SNPs (A^637^G^638^G^639^) of the Assaf growth hormone have been confirmed in the result of the multiple alignments comparing with of all breeds in the GenBank database. Ossimi sheep have three unique SNPs (A^12^A^13^G^14^) encodes for lysine (Lys^2^, K) *vs.* alanine (Ala^3^, A) in all GenBank database. Due to the difference of physicochemical properties of Lys and Ala residues [27], therefore substitution of Ala with Lys in Ossimi sheep may have an adverse effect on the function of growth hormone and thereby, affect animal performance.

Ossimi, Afghani, and Kazakh sheep are the most breeds have SNPs compared to the Genbank database that may be due to the interfering of random crossbreeding in each breed. There are two alleles substitutions in Ossimi and Afghani sheep which yield a new codon that encodes for different amino acid; for Ossimi sheep, Gg^472^c^473^ encodes for glycine (Gly, G), and Ca^628^g^629^ encodes for glutamine (Gln, Q) for Afghani sheep, respectively compared to GTT for valine (Val, V) and CGC for arginine (Arg, R) in the GenBank database and in Afghani sheep, Gg^389^c^390^ for glycine (Gly, G) compared to GTT for valine (Val, V) in the GenBank database. Both glycine and glutamine did not have the same physicochemical properties of valine and arginine; thereby, substitution between them may harm the protein function [27].

#### Multiple alignments of Bo_GH sequence vs. GenBank goat database

Pakistani Kamori, Tharri, Beetal, and Indian Beetal goat are the most breeds have SNPs compared to the GenBank goat database. Most goat breeds in Pakistani or in India are the multipurpose type used for meat and milk production or sometimes also for hair [28]. This may explain the variety of SNPs in the growth hormone cDNA sequence in these breeds due to the random crossbreeding in each breed.

Pakistani Kamori, Tharri, and Beetal goat shared with the same SNPs encode for the same amino acids. These single nucleotide polymorphisms can be used to develop genetic markers for each breed. Single nucleotide polymorphisms of A/G was observed in Afghani sheep (A) and Indian Beetal goat vs. G in GenBank sheep and goat database. In Pakistani Beetal goat has G/A SNP; g^358^GC (Gly^120^) compared to A^78^GC (Ser^120^) in GenBank goat database. This substitution of Gly with Ser is difficult to predict, and modeling studies suggest that it would not be great [18]. The multiple alignments of the growth hormone of sheep and goats in the present results are in agreement with the results reported previously, where there were two genotypes, G/G nucleotide and A/G nucleotide, in sheep and goat [29, 30]. This A/G genotype is associated with high growth traits like birth chest girth, weaning weight, large litter sizes, and good body conformation. It may be due to the presence of both glycine and serine residues in the heterozygous animals, and these residues are interconvertible; when the body needs any of them and not available from the feed, it uses serine to produce glycine and vice versa. The serine residue is involving in the metabolic processes that burn glucose and fatty acids for energy, besides that used to make creatine which combines with water to “pump up” muscle mass.

Moreover, glycine is required for the synthesis of protein, construction of DNA, as well as RNA and synthesis of bile acids and other amino acids in the body, and it helps in retarding degeneration of muscles [31]. Therefore, production improvements can be achieved using these growth hormone SNPs through the selection of animals that have AG genotype and enter them in breeding programs of Egyptian sheep and goat as a way to increase their productivity. In Egyptian sheep and goat, SNP of g^55^G and a^55^G with two genotypes (G/G and A/G) has detected in the growth hormone sequence of Egyptian sheep and goat. The GG and AG genotype frequencies were 35.56 and 64.44% in Barki sheep, 19.23 and 80.77% in Rahmani sheep, and 76.67 and 23.33% in Ossimi sheep, respectively. In goats, the GG and AG genotype frequencies were 0 and 100% for Baladi, 13.33 and 86.67% for Barki, and 23.53 and 76.47% for Zaraibi, respectively [32].

In the present study, the multiple alignments showed that SNP of C/T was observed in both sheep and goat; TCc^248^ (Ser^80^) in Ossimi sheep and TCc^254^ (Ser^81^) in China sheep vs. TCC^251^ (Ser^81^) in all GenBank sheep database, while TCc^246^ and TCc^168^ for Ser^81^ in Indian Tibetan, Lazhi, and Beetal goats vs. TCT^263^ for Ser^81^ in for all GenBank goat database. The present results are confirmed previously by [33], where the heterozygote counterparts for C^1763^T and A^1780^G SNPs in GH gene sequence exhibited heavy body weights (*p* < 0.05) in Indian Osmanabadi and Sangamneri goat breeds.

### The prediction of growth hormone promoter

The promoter controls and regulates the first step of gene expression, so, is the most important regulatory sequence in the gene [34]. The promoter sequence in As_GH and Bo_GH sequence was completely identical sequence and has the same length irrespective of position difference.

### Protein structure annotation

#### Signal peptides sequence

The signal peptides are unique sequence and usually ranged from 16 to 30 residues extended in the N-terminal of newly synthesized secretory and involved in the transport of the protein to or via cell membranes and targeting to the endoplasmic reticulum (ER) membrane for translation initiation [35, 36]. The signal peptides are discarded during protein transportation via cell membrane by a specific peptidase [37]; they are consisting of tripartite structure (1) region of hydrophilic residues, (2) region of hydrophobic residues, and (3) cleavage site with signal peptidase (SPase) [37].

#### Conserved domain and motifs

In the conserved domain of sheep breeds, residues of Ser, Gly, and Glu have the same physicochemical properties; therefore, the substitution between them may not affect the protein function [27]. Likewise, in the conserved domain of the goat breeds, the residues of Ser, Gly, and Leu, Val have the same physicochemical properties. Therefore, the substitution between them may not affect the protein function [27]. Other residues substitutions in both conserved domain may affect protein function due to the difference in physicochemical properties [27].

The GH protein sequence is strongly conserved in most mammals, but there are differences in the biological and receptor-binding properties due to the species-specificity of receptor-binding [38]. A protein domain is a conserved and distinct part of molecular evolution, usually related to specific molecular functions of such protein folding and can function independently of the rest of the protein. Detection of the significant protein conserved domains is often required for basic cellular function, stability, or reproduction [39]. Detection of the conserved domain on the growth hormone sequence of Assaf sheep and Boer goat may be indicated that the isolation and sequencing processes of growth hormone are achieved correctly.

The protein motifs are consecutive and conserved amino acids sequence of protein families (called motifs signatures) and can often be used as a prediction tool for protein function [40]. Therefore, the Bo_GH sequence protein and all sheep and goat breeds in GenBank database had two common motifs signature: Somatotropin_1 (CFSETIPAPTGKNEAQQKSDLELLRI SLLLIQSW) and Somatotropin_2 (CFRKDLHKTETYLRVMKC). Any change in these consecutive protein residues causes an inability to predict the motif signatures. Interestingly, the As_GH protein sequence had only one motif signature, Somatotropin_1 (CFSETIPAPTGKNEAQQKSDLELLRISLLLIQSW), and the second motif signature (Somatotropin_2) is unpredictable. Three novel distinct nucleotides (AAG) that encode for arginine (R^194^) in protein sequence were observed inside the consecutive sequence of the second motif signature_2 (CFRK**R**DLHKTETYLRVMKC). Therefore, due to the presence of this insertion mutation makes it undetectable. There are not available growth hormone cDNA sequences for Assaf sheep in the GenBank database; therefore, it could not confirm that this mutation is specific for this breed, or this is due to individual mutation. Further studies are needed to confirm that these SNPs are stable mutations in Assaf sheep breed or are transiently mutation.

#### Cysteine bridge and disulfide bonds

The disulfide bonds play a crucial role in the folding and stability of most extracellular secreted proteins [41], protection of protein integrity from the extracellular milieu oxidants and proteolytic enzymes, thereby increase their half-life of the protein [42]. Through the oxidative folding process, four from five cysteine residues are establishing two disulfide bonds between the thiol groups of cysteine residues to stabilize the folded form of a protein [43]. Also, in the intracellular environment, the sulfhydryl side chain of cysteines is excellent for binding to metals, such as zinc [44].

### Protein alignments of As_GH and Bo_GH predicted protein sequence

#### Pairwise alignments

As_GH protein sequence has a unique residue (arginine, R^194^) that was absented in the Bo_GH protein sequence. Arginine (Arg) is a polar and positively charged amino acid; it prefers to be on the surface of the protein and frequently involved in salt-bridges where they pair with a negatively charged residue (aspartate or glutamate) to create stabilizing hydrogen bonds. Therefore, the presence of arginine (insertion mutation) in the As_GH sequence may be important for increasing the protein stability [45] and also may be involved in the growth hormone-receptor binding [46].

#### Multiple alignments of growth hormone protein residues

Gene families arise by gene duplication and natural selection. The multiple alignments are an essential study to understand the evolutionary event between species [47]. In most mammals, the GH sequence is strongly conserved, but differences in the biological and receptor-binding properties are due to the species-specificity of receptor-binding [38]. Due to the difference of physicochemical properties between Lys and Ala [27], the substitution of Lys^2^ residue in the GH signal peptide of Ossimi sheep vs*.* Ala^3^ residue in the GenBank database may be causing a reduction in GH secretion in Ossimi sheep. Although there is a difference in the properties of Pro^6^ residue (small residue) and Thr^5^ residue (polar residue), they shared in to facilitate the intracellular signal transduction of the proteins, so they can be substituted without negative effect on protein function [9, 48, 49].

The sheep GenBank database has dominant SNP at AC^526^A that encoded for Thr^173^ compared to Ag^518^A encoded for Arg^173^ in Chines Tibetan sheep. The SNP of C in the growth hormone sequence showed a positive association with the growth rate [50]. Therefore, substation C to G may have a negative effect on the growth rate of Chines Tibetan sheep.

The substitution of Val^156^ with Gly^156^ and Gly^35^ with Ser^5^ in Afghani sheep breed may be not affecting GH function because the Gly and Val residues are small size and hydrophobic residues, and Gly and Ser residues are tiny and small residues; hence, the substitution is functional [49]. Likewise, the substitution of Glu^154^ residue that found in all breeds with Gly^154^ in Tibetan sheep breed may be causing a reduction in growth hormone function due to the differences between them in physiochemical properties [48], where the Gly is a small and hydrophobic residue, while the Glu is a polar and negative charged residue [51]. The Tyr^168^ residue is presented in the growth hormone protein sequence of GenBank sheep breeds vs. His^168^ residue in Pakistani Latti sheep breed. Tyrosine and histidine are polar amino acids (neutral), hence may the Tyr residue can be substituted with His residue without the adverse effect of GH protein function [48].

Pakistani goat breeds (Tharri, Kamori, and Beetal) were shared in the same residue substitution of Ala^15^ vs. Thr^15^ in GenBank goat breeds database; this substitution may be not affecting the GH protein function because both residues are small size [49], and this may be used this mutation as a genetic marker for these goat breeds. The substitution of Gly^156^ Pakistani Kamori and Beetal goat breeds with Val^156^ in GenBank goat breeds database may be acceptable where the Gly and Val residues are small sizes and hydrophobic residues; hence, the substitution is functional [49, 51]. The leucine and proline are very non-reactive residues and rarely directly involved in protein active or binding sites. Leucine can be substituted by other hydrophobic, particularly aliphatic, residues. Since the leucine and proline are small sizes and aliphatic residues, so leucine can be substituted by proline residue in Lazhi and Tharri goat [48, 51].

## Conclusion

It was concluded that the GH sequences of Assaf and Boer goat are highly conserved and the homogeny in the codon region (99.5%). The Assaf sheep has a unique three SNPs (A^637^A^638^G^639^) that encodes for arginine (Arg^194^) that absented in growth hormone cDNA sequences of Boer goat in the current study and GenBank database breeds. Since there were no available records of GH cDNA sequences of Egyptian sheep or goats in the GenBank database, it was not possible to confirm that these SNPs that were reviewed in the current study are a distinctive characteristic for Egyptian breeds. Therefore, further studies are needed to analyses the genetic variations of growth hormone gene in different sheep and goat breeds in Egypt as well as documenting the relationship between these variations and the productive performance of animals.

## Data Availability

Not applicable

## Abbreviations

GH: Growth hormone
AC: Accession number
As_GH: Assaf sheep
Bo_GH: Boer goat
CDS: Complete coding sequences
NCBI: National Center of Biotechnology Information
PCR: Polymerase chain reaction

## References

[1] Zi XD, Mua XK, Wang Y (2013). Variation in sequences and mRNA expression levels of growth hormone (GH), insulin-like growth factor I (IGF-I) and II (IGF-II) genes between prolific Lezhi black goat and non-prolific Tibetan goat (Capra hircus). Gen Comp Endocrinol.

[2] Francis SM, Veenvliet BA, Littlejohn RP, Stuart SK, Suttie JM (1995). Growth hormone (GH) secretory patterns in genetically lean and fat sheep. Proc N Z Soc Anim Prod.

[3] Pollott GE, Gootwine E (2004). Reproductive performance and milk production of Assaf sheep in an intensive management system. J Dairy Sci.

[4] Gootwine E (2011). Mini review: breeding Awassi and Assaf sheep for diverse management conditions. Trop Anim Health Prod.

[5] Casey N, Van Niekerk HWA (1988). The Boer goat. I. Origin, adaptability, performance testing, reproduction and milk. Small Rumin Res.

[6] Erasmus JA (2000). Adaptation to various environments and resistance to disease of the Improved Boer goat. Small Rumin Res.

[7] Malan SW (2000). The improved Boer goat. Small Rumin Res.

[8] Hall TA (1999). BioEdit: a user-friendly biological sequence alignment editor and analysis program for Windows 95/98/NT. Nucleic Acids Symp Ser.

[9] Artimo P, Jonnalagedda M, Arnold K, Baratin D (2012). SIB bioinformatics resource portal. Nucleic Acids Res.

[10] Thomas NP, Søren B, Gunnar VH, Henrik N (2011). SignalP 4.0: discriminating signal peptides from transmembrane regions. Nat Methods.

[11] Omasits U, Ahrens CH, Müller S, Wollscheid B (2014). Bioinformatics.

[12] Ferre F, Clote P (2005). Disulfide connectivity prediction using secondary structure information and diresidue frequencies. Bioinformatics.

[13] Reese MG (2001). Application of a time-delay neural network to promoter annotation in the Drosophila melanogaster genome. Comput Chem.

[14] Marchler-Bauer A, Bryant SH (2011). CD-Search: protein domain annotations on the fly. Nucleic Acids Res.

[15] Lucy M, Hauser SD, Eppard PJ, Krivi GG, Clark JH, Bauman DE, Collier RJ (1991). Genetic polymorphism within the bovine somatotropin (bST) gene detected by polymerase chain reaction and endonuclease digestion. J Dairy Sci.

[16] Schlee P, Graml R, Rottmann D, Pirchner F (1994). Influence of growth-hormone genotypes on breeding values of Simmental bulls. J Anim Breed Genet.

[17] Ge W, Davis ME, Hines HC, Irvin KM, Simmen RCM (2003). Association of single nucleotide polymorphisms in the growth hormone and growth hormone receptor genes with blood serum insulin-like growth factor I concentration and growth traits in Angus cattle. J Anim Sci.

[18] Wallis M, Lioupis A, Wallis OC (1998). Duplicate growth hormone genes in sheep and goat. J Mol Endocrinol.

[19] Bastos E, Cravador A, Azevedo J, Guedes H (2001) Single strand conformation polymorphism (SSCP) detection in six genes in Portuguese indigenous sheep breeds Churra da Terra Quente. Biochtechnol Agron Soc Environ (5):7–15

[20] Gupta N, Ahlawat SPS, Kumar D, Gupta SC, Pandey A, Malik G (2007). Single nucleotide polymorphism in growth hormone gene exon-4 and exon-5 using PCR-SSCP in Black Bengal goats: a prolific meat breed of India. Meat Sci.

[21] Dybus A (2002). Associations between Leu/Val polymorphism of growth hormone gene and milk production traits in black-and-white cattle. Archiv Tierzucht.

[22] Beauchemin VR, Thomas MG, Franke DE, Silver GA (2006). Evaluation of DNA polymorphisms involving growth hormone relative to growth and carcass characteristics in Brahman steers. Genet Mol Res.

[23] Katoh K, Kouno S, Okazaki A, Suzuki K, Obara Y (2008). Interaction of GH polymorphism with body weight and endocrine functions in Japanese black calves. Domest Anim Endocrinol.

[24] Marques MDR, Santos IC, Carolino N, Belo CC, Renaville R, Cravador A (2006). Effects of genetic polymorphisms at the growth hormone gene on milk yield in Serra da Estrela sheep. J Dairy Res.

[25] Malveiro E, Pereira M, Marques PX, Santos IC, Belo C, Renaville R, Cravador A (2001). Polymorphisms at the five exons of the growth hormone gene in the algarvia goat: possible association with milk traits. Small Rumin Res.

[26] Boutinaud M, Rousseau C, Keisler DH, Jammes H (2003). Growth hormone and milking frequency act differently on goat mammary gland in late lactation. J Dairy Sci.

[27] Xidan L, Marcin K, Xia S, Muhammad A, Örjan C, Stefan M (2013) PASE:a novel method for functional prediction of amino acid substitutions based on physicochemical properties. Front Genet Bioinform Comput Biol (4):1–6 10.3389/fgene.2013.00021 PMC3589708 23508070

[28] Nazeer M, Shah SH (2018). Morphological characterization of indigenous goats breeds of Khyber Pakhtunkhwa, Pakistan. Sarhad J Agric.

[29] Hua GH, Chen SL, Yu JN, Cai KL, Wu CJ (2009). Polymorphism of the growth hormone gene and its association with growth traits in Boer goat bucks. Meat Sci.

[30] Zhang C, Liu Y, Huang K, Zeng W, Xu D, Wen Q, Yang L (2011). The association of two single nucleotide polymorphisms (SNPs) in growth hormone (GH) gene with litter size and superovulation response in goat-breeds. Genet Mol Biol.

[31] Lehninger AL, Nelson DL, Cox MM (2005). Lehninger principles of biochemistry.

[32] Othman EO, Sally SA, Heba AMA, Omaima MA (2015). Genotyping of growth hormone gene in Egyptian small ruminant breeds. Biotechnology.

[33] Wickramaratne SHG, Ulmek BR, Dixit SP, Kumar S, Vyas MK (2010). Use of growth hormone gene polymorphism in selecting Osmanabadi and Sangamneri goats. Trop Agric Res.

[34] Carey M, Smale ST (2000). Transcriptional regulation in eukaryotes: concepts, strategies, and techniques.

[35] Duffaud GD, Lehnhardt SK, March PE, Inouye M, Knauf PA, Cook JS (1985). Structure and function of the signal peptide. Membrane protein biosynthesis andturnover.

[36] Kober L, Zehe C, Bode J (2013). Optimized signal peptides for the development of high expressing CHO cell lines. Biotechnol Bioeng.

[37] Nakai K (2000). Protein sorting signals and prediction of subcellular localization. Adv Protein Chem.

[38] Lioupis A, Wallis OC, Wallis M (1997). Cloning and characterisation of the gene encoding red deer (Cervus elaphus) growth hormone: implications for the molecular evolution of growth hormone in artiodactyls. J Mol Endocrinol.

[39] Thompson JD, Gibson TJ, Plewniak F, Jeanmougin F, Higgins DG (1997). The CLUSTAL_X windows interface: flexible strategies for multiple sequence alignment aided by quality analysis tools. Nucleic Acids Res.

[40] Peer B, Eugene VK (1996). Protein sequence motifs. Curr Opin Struct Biol.

[41] Paladini AC, Pena C, Poskus E (1983). Molecular biology of growth hormone. Crit Rev Biochem.

[42] Hogg PJ (2003). Disulfide bonds as switches for protein function. Trends Biochem Sci.

[43] Wedemeyer WJ, Welker E, Narayan M, Scheraga HA (2000). Disulfide bonds and protein folding. Biochemistry.

[44] Barnes MR, Russell R (1999). BA lipid-binding domain in Wnt: a case of mistaken identity?. Curr Biol.

[45] Copley RR, Barton GJ (1994). A structural analysis of phosphate and sulphate binding sites in proteins. Estimation of propensities for binding and conservation of phosphate binding sites. J Mol Biol.

[46] Chêne N, Martal J, de la Llosa P, Charrier J (1989). Growth hormones. II. Structure–function relationships. Reprod Nutr Dev EDP Sci.

[47] Iwatsuki K, Oda M, Sun W, Tanaka S, Ogawa T, Shiota K (1998). Molecular cloning and characterization of a new member of the rat placental prolactin (PRL) family, PRL-like protein H. Endocrinology.

[48] Betts MJ, Russell RB, Barnes MR (2003). Amino acid properties and consequences of substitutions. Bioinformatics for geneticists.

[49] Ng PC, Henikoff S (2003). SIFT: predicting amino acid changes that affect protein function. Nucleic Acids Res.

[50] Hediger R, Johnson SE, Barendse W (1990). Assignment of the growth hormone gene locus to 19q26-qter in cattle and to 11q25-qter in sheep by in situ hybridization. Genomics.

[51] Ng PC, Henikoff S (2001). Predicting deleterious amino acid substitutions. Genome Res.

